# Supplementary material for: Adenosine, Schizophrenia and Cancer: Does the Purinergic System Offer a Pathway to Treatment?
Source: Int J Mol Sci. 2022 Oct 5;23(19):11835. doi: 10.3390/ijms231911835 (PMC9570456; doi:10.3390/ijms231911835)
Supplement: Supplementary file 1 [file ijms-23-11835-s001.zip › ijms-1884039-supplementary.pdf]

Supplementary Figure 1: Small molecule structures for adenosine system modulators identified in Table 3

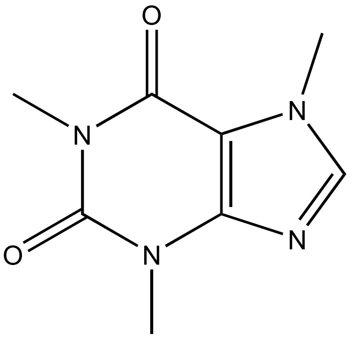

caffeine

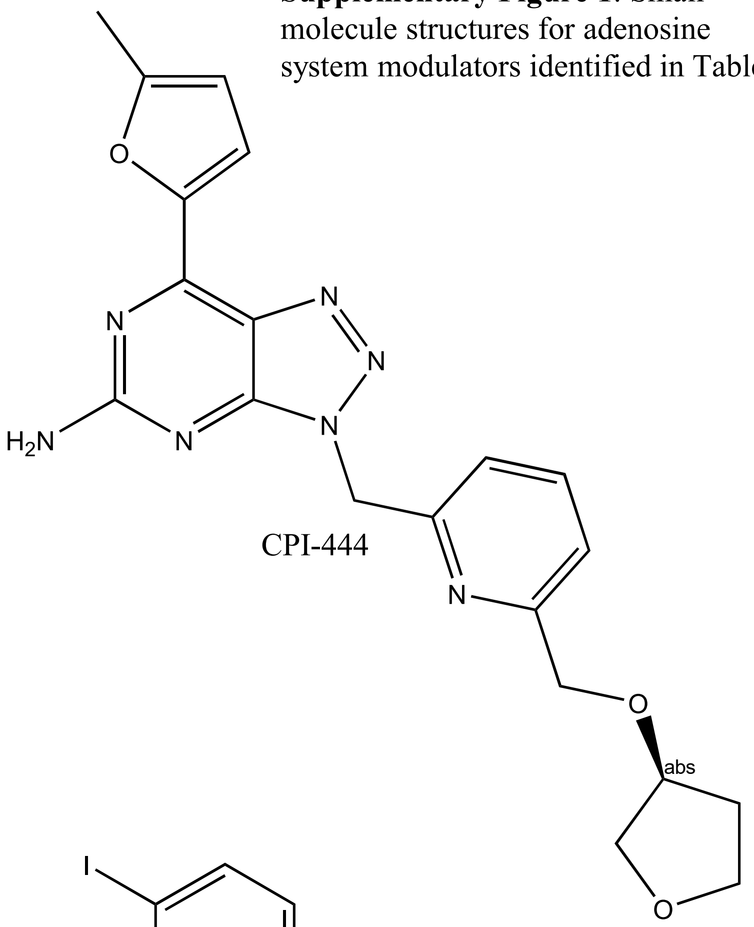

CPI-444

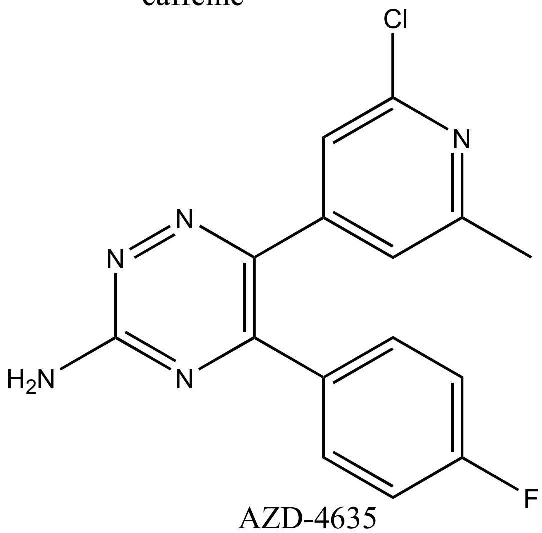

AZD-4635

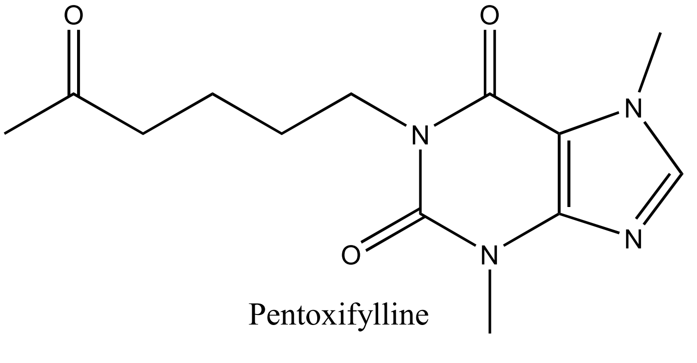

Pentoxifylline

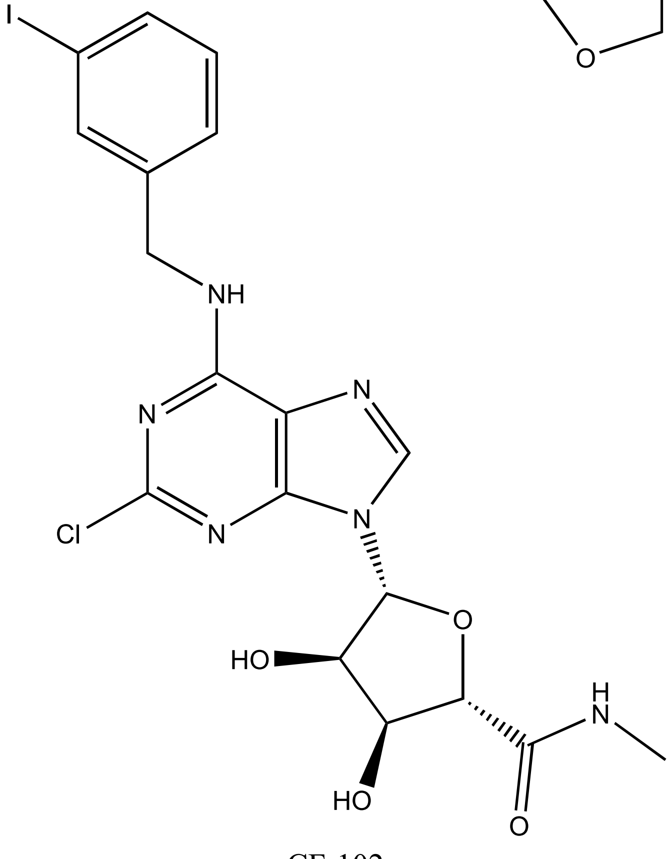

CF-102

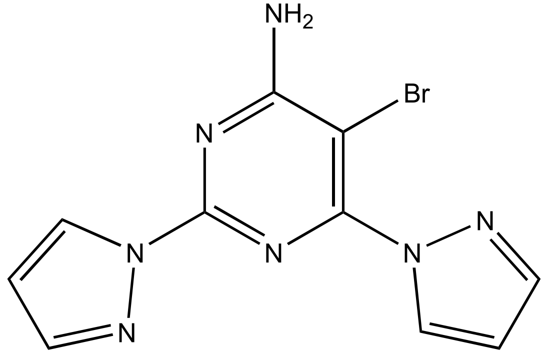

NIR-178

Supplementary Table 1: Purinergic receptors. Nomenclature, associated ligands, G-proteins, and ions.

**Protein name**	**Gene Name**	**Ligand**	**G-protein**	**Ions**
Adenosine receptor A_1	ADORA1	Adenosine	G_i and G_o	
Adenosine receptor A_{2A}	ADORA2A	Adenosine	G_s^c, G_{olf}	
Adenosine receptor A_{2B}	ADORA2B	Adenosine	G_s^c	
Adenosine receptor A_3	ADORA3	Adenosine	G_{i3}, G_q	-
P2X purinoceptor 1	P2RX1	ATP	$G_{\alpha q}$	Na^+, K^+, Ca^{2+}
P2X purinoceptor 2	P2RX2	ATP	$G_{\alpha q}$	Na^+, K^+, Ca^{2+}
P2X purinoceptor 3	P2RX3	ATP	$G_{\alpha q}$	Na^+, K^+, Ca^{2+}
P2X purinoceptor 4	P2RX4	ATP	$G_{\alpha q}$	Na^+, K^+, Ca^{2+}
P2X purinoceptor 5	P2RX5	ATP	$G_{\alpha q}$	Na^+, K^+, Ca^{2+}
P2X purinoceptor 6	P2RX6	ATP	$G_{\alpha q}$	Na^+, K^+, Ca^{2+}
P2X purinoceptor 7	P2RX7	ATP	$G_{\alpha q}$	Na^+, K^+, Ca^{2+}
P2Y purinoceptor 1	P2RY1	ADP	G_q	
P2Y purinoceptor 2	P2RY2	ATP/UTP	$G_{q, o, 12}$	
P2Y purinoceptor 4	P2RY4	UTP	$G_{q,o}$	
P2Y purinoceptor 6	P2RY6	UDP	$G_{q, 12/13}$	-
P2Y purinoceptor 11	P2RY11	ATP	$G_{q, s}$	
P2Y purinoceptor 13	P2RY13	ADP	$G_{i/o}$	
P2Y purinoceptor 14	P2RY14	UPD/UDP-glucose		

Purinergic receptor ligands, ions, and G-proteins adapted from ref [60-68]
